# Effect of a cannabidiol-based mouthwash on dental enamel properties and biofilm control: an In situ study

**DOI:** 10.1007/s00784-026-06985-7

**Published:** 2026-07-01

**Authors:** Anna Luísa Araujo Pimenta, Lucas Andrade de Sousa, Carlos Henrique Gomes Martins, Fernanda de Carvalho Panzeri

**Affiliations:** 1 Department of Pediatric Dentistry, School of Dentistry of Ribeirao Preto, University of São Paulo, Ribeirao Preto, Brazil; 2 Institute of Biomedical Sciences (ICBIM), Federal University of Uberlândia (UFU), Uberlândia, Brazil; 3 Department of Dental Materials and Prosthodontics, Ribeirão Preto School of Dentistry, University of São Paulo, Av. do Café, s/n, Ribeirão Preto, SP 14040-904 Brazil

**Keywords:** Cannabidiol, Mouthwash, Cariogenic challenge, *In Situ*

## Abstract

**Objectives:**

This study evaluated the antibiofilm activity of experimental mouthwash containing different concentrations of cannabidiol (CBD) and the in situ effects on the physical and mechanical properties of dental enamel.

**Methods:**

Bovine enamel fragments (6 × 6 × 2 mm) were mounted in intraoral appliances worn by 14 participants in a crossover design. Mouthwash containing CBD (0%, 0.01%, 0.05%, and 0.1%) and 0.12% chlorhexidine (CHX) were tested. Each experimental phase lasted 7 days, separated by washout periods. One side of the appliance was exposed to a cariogenic challenge (20% sucrose) prior to treatment. Surface roughness (Ra), microhardness (%KHN), and color change (ΔE00) were measured before and after treatments. Biofilm and yeast counts (log10 CFU) were quantified, and enamel surfaces were analyzed by scanning electron microscopy. Data were analyzed using two-way ANOVA with Bonferroni post hoc tests and Kruskal–Wallis with Dunn’s test (*P* < 0.05).

**Results:**

Sucrose did not significantly affect Ra (*P* > 0.05), although CBD 0.1% showed higher roughness than CHX under sucrose exposure (*P* < 0.05). No significant differences in %KHN were observed among treatments; however, sucrose reduced microhardness in the placebo and CBD 0.01% groups (*P* < 0.05). CHX exhibited the highest ΔE00 values (*P* < 0.05). Biofilm formation was similar among CHX, CBD 0.05%, and CBD 0.1% (*P* > 0.05), while CHX showed lower yeast counts than CBD 0.01% and CBD 0.1% (*P* < 0.05).

**Conclusion:**

CBD 0.05% demonstrated potential for biofilm control without adversely affecting enamel properties.

**Clinical relevance:**

This study provides evidence supporting a natural compound–based mouthwash as a clinically viable alternative to chlorhexidine, showing similar efficacy and no associated adverse effects under the conditions tested.

## Introduction

Oral health is essential for overall well-being, directly influencing an individual’s ability to speak, eat, smile, and engage in social interactions without pain or discomfort. Therefore, maintaining good oral health is fundamental to quality of life [1, 2]. Nevertheless, oral diseases remain a significant global public health concern. Among the 50 most prevalent oral conditions analyzed in the Global Burden of Disease study from 1990 to 2017, dental caries stands out as the most widespread. It is estimated that approximately 2.3 billion people worldwide have untreated caries in permanent teeth [2–4].

Dental caries is a biofilm-mediated disease associated with frequent sugar exposure, leading to an imbalance between demineralization and remineralization processes in dental tissues [4]. Sucrose is one of the main carbohydrates involved in biofilm formation. This disaccharide, composed of glucose and fructose linked by a glycosidic bond, provides a readily available energy source for microbial metabolism [5, 6].

Mechanical methods for biofilm control are well established and effective. However, there is a growing interest in adjunctive agents capable of controlling microbial biofilm without altering the dental enamel surface [7]. In this context, mouthwashes have been widely used as an auxiliary strategy, particularly for patients with limited ability to maintain adequate oral hygiene, such as elderly or medically compromised individuals [8, 9].

Chlorhexidine digluconate (CHX) has been considered the gold standard mouthwash in dentistry for several decades [10, 11]. It is a cationic bisbiguanide with bacteriostatic activity at low concentrations and bactericidal effects at higher concentrations. However, its long-term use has been associated with several adverse effects, including taste alteration, tooth and tongue staining, oral mucosal irritation, parotid gland swelling, xerostomia, and the potential development of antimicrobial resistance [9, 12–14]. These limitations highlight the need to investigate alternative therapeutic agents [9, 12, 14, 15].

In this scenario, the incorporation of natural compounds into oral care products has gained increasing attention [16, 17]. Cannabidiol (CBD), a non-psychoactive compound derived from *Cannabis sativa*, has attracted considerable interest due to its favorable safety profile and its anti-inflammatory, immunomodulatory, and antimicrobial properties against pathogenic microorganisms.

Previous studies have demonstrated the antimicrobial activity of cannabidiol against oral pathogens and Gram-positive bacteria; however, its specific effects on cariogenic species such as *Streptococcus mutans* and *Lactobacillus spp.* remain limited and require further investigation [18–21]. Furthermore, the literature emphasizes the need for additional studies under clinically relevant conditions to better understand the effectiveness of CBD-based products.

 In situ models are essential for evaluating the influence of oral environmental factors, such as temperature, humidity, and biofilm dynamics, on the performance of therapeutic agents over time [21]. Additionally, it is crucial to investigate the effects of these agents on the physical and mechanical properties of dental enamel [21, 22].

Therefore, this study aimed to evaluate, in situ, the antibiofilm potential of cannabidiol-containing mouthwash at different concentrations and their effects on the physical and mechanical properties of dental enamel. Specifically, enamel surface roughness, Knoop microhardness, color stability, and morphological characteristics were assessed, as well as the ability of these formulations to control biofilm formation. The null hypothesis tested was that cannabidiol-based mouthwashes, regardless of concentration, would not exhibit greater antimicrobial activity than the positive control (0.12% chlorhexidine) and would not promote significant changes in enamel color, surface roughness, or microhardness compared to the positive control.

## Materials and methods

This study was conducted following an in situ, double-blind, crossover design, consisting of five experimental phases, each lasting 7 days, with 7-day washout intervals between phases to eliminate potential residual effects from previous treatments [23–25]. The study protocol was approved by the Institutional Human Research Ethics Committee (CAAE No. 79550324.6.0000.5419).

Bovine enamel fragments (6 mm × 6 mm × 2 mm) were obtained using a precision cutting machine (Isomet 1000, Buehler, Lake Bluff, IL, USA). The enamel surface was flattened and polished using a mechanical polishing machine (Polipan-U, Panambra, São Paulo, SP, Brazil) with silicon carbide abrasive papers (600, 1200, and 2000 grit), under low-speed conditions and water irrigation. Surface roughness (Ra) was standardized in 0.2 μm before the treatments.

### Surface roughness assessment

Surface roughness of the enamel specimens was measured using a profilometer (SJ-201P, Mitutoyo, Tokyo, Japan) over a 5 mm path, with three cut-off lengths of 0.8 mm and a scanning speed of 0.5 mm/s. For each specimen, three readings were taken at different locations on the enamel surface: one at the center, one 1 mm to the right, and one 1 mm to the left. The average of these three readings was considered the baseline surface roughness value (Ra) for each specimen. Only specimens presenting a surface roughness lower than 0.2 μm were included in the study.

### Knoop microhardness measurement

Knoop microhardness (KHN) values were measured using a microhardness tester (HMV-2, Shimadzu, Tokyo, Japan). A vertical load of 25 g was applied to the enamel surface for 5 s, producing a diamond-shaped indenter with an elongated rhomboidal base indentation. Five indentations were performed on each specimen: one at the center, and four additional indentations positioned 1 mm to the right and left of the central mark, both above and below it. KHN was calculated by measuring the length of the longest diagonal of the indentation and applying the following formula:

$$\:\mathrm{K}\mathrm{H}\mathrm{N}=\mathrm{1,451}\:\mathrm{F}/\mathrm{d}^2$$

Where:

KHN = Knoop hardness number.

F = applied load (25 g).

d = length of the longest diagonal of the indentation (mm). The average of the five measurements was considered the baseline KHN value for each specimen.

### Color measurement

Baseline color measurements were obtained using a spectrophotometer (VITA Easyshade, VITA Zahnfabrik, Bad Säckingen, Germany) inside a light chamber (Gester International, Fujian Province, China) featuring a neutral gray background (Munsell N-7). The device is equipped with a digital tip that emits a standardized light through optical fibers that capture the reflected light from the enamel surface and generate chromatic coordinates according to the CIE L*a*b* color system.

The primary standard illuminant used was D65, which simulates daylight. Samples were placed on a standardized white background (White Standard Sphere for 45º, 0º Reflectance, Gardner Laboratory Inc., Bethesda, Geretsried, Germany). To standardize the color analysis, three repeated measurements were performed and the average of these readings was considered the initial color values. Readings presenting variations greater than 1.0 unit in L*, a*, or b* values were discarded, and new measurements were obtained to ensure accuracy.

### Participant recruitment

Fourteen volunteers (*n* = 14) were enrolled in the study. The sample size was based on previous in situ investigations [23, 24, 26], which recommended the inclusion of 10 to 12 participants. A larger number of volunteers was recruited to compensate for potential dropouts during the experimental period. Fourteen volunteers were enrolled in the study and 13 completed all five experimental phases (*n* = 13). The study was therefore analyzed on a per-protocol basis, with the full sample of 14 participants included in all statistical analyses.

### Inclusion criteria

Participants were required to meet the following criteria:

- Age between 20 and 40 years.
- Agreement to participate in the study, confirmed by signing a written informed consent form;
- Good general health status;
- Absence of active caries lesions or periodontal disease.

### Initial exclusion criteria

Participants were excluded if they presented:

- Use of medications or systemic conditions affecting salivary flow;
- Use of antibiotics or tobacco;
- Use of removable dental prostheses (partial or complete) or orthodontic appliances;
- Pregnancy or breastfeeding.

### Exclusion criteria during the experimental period

Participants were excluded during the study in cases of:

- Voluntary withdrawal;
- Changes in health status resulting in altered salivary flow or the need for antibiotic therapy.

### Fabrication and installation of the intraoral appliance

**Fig. 1:**
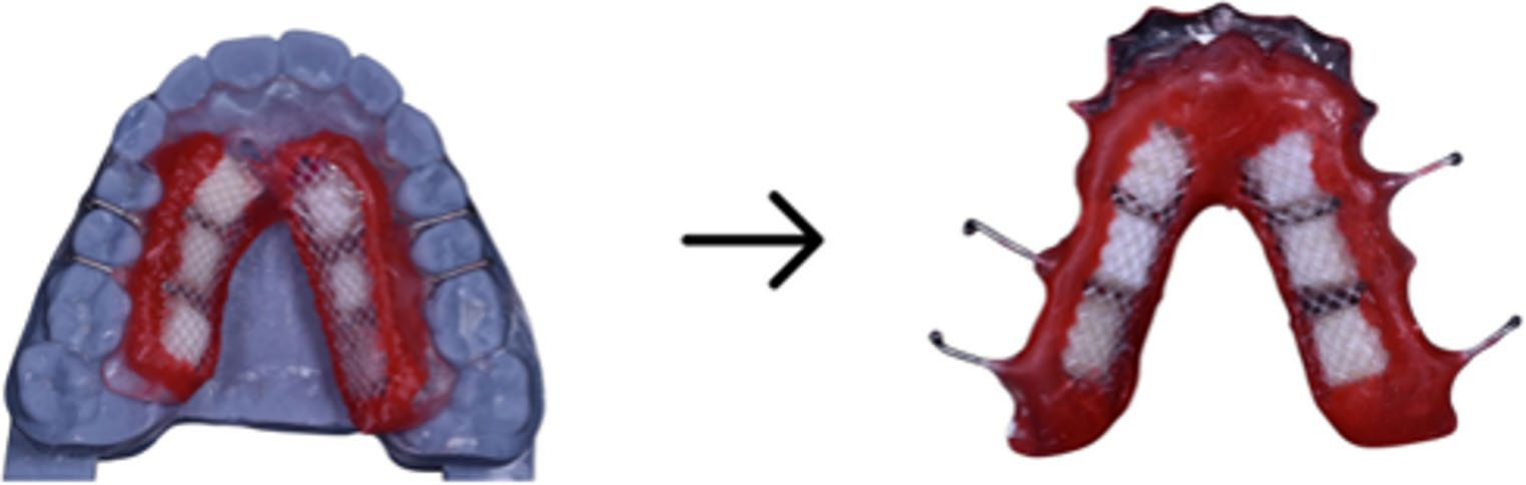
Intraoral palatal appliance used in the in situ experimental model, containing bovine enamel specimens for biofilm formation and treatment exposure

An intraoral scan of the arches of each participant was obtained using a digital Scanner (Primescan, Dentsply Sirona, São Paulo, SP, Brazil). Three-dimensional printed models were fabricated using a 3D printer (Photon Mono SE, Anycubic, Shenzhen, China) and 3D printing UV sensitive resin (Anycubic, Shenzhen, China, UV wavelength 405 nm). Based on these models, intraoral appliances were fabricated using heat-polymerized acrylic resin (Clássico Produtos Odontológicos Ltda., São Paulo, SP, Brazil), with a thickness of 0.3 mm to enhance participant comfort during the experimental phases (Fig. 1).

Each intraoral appliance contained six bovine enamel specimens (three on each side of the device). Prior to placement, the specimens were sterilized in a 2% formaldehyde solution (pH 7.0) for 1 month, according to a previously described protocol [26]. The enamel fragments were carefully fixed to the intraoral appliance using sticky wax (Kota, São Paulo, SP, Brazil), positioning the enamel surface facing outward toward the oral environment.

A polyethylene mesh was positioned over the enamel specimens using sticky wax, leaving a standardized 1-mm gap between the mesh and the enamel surface [27]. This setup allowed for undisturbed biofilm accumulation on the enamel surface during the experimental period. The intraoral appliances were delivered to the participants, who were instructed to wear them continuously throughout each experimental phase, removing them only during meals and for the application of the assigned treatments. In addition to the appliance, participants received a kit containing a soft-bristled toothbrush (Condor, São Bento do Sul, SC, Brazil) and a fluoridated dentifrice (Colgate Total 12, Colgate-Palmolive Ind. Ltda., São Bernardo do Campo, SP, Brazil). Detailed instructions were provided regarding toothbrushing procedures, appliance hygiene, and the application protocol for the experimental treatments.

### Preparation of experimental solutions

Cannabidiol-containing mouthwashes were formulated with a humectant, surfactant, sweetener, flavoring agent, excipient, preservative, and water, containing CBD at concentrations of 0.01%, 0.05%, or 0.1%. CBD was provided as an oil-based isolate derived from Cannabis sativa L. and obtained by alcoholic extraction (Tegra Usaline Isolate 6,000 mg, TegraPharma, Ocoee, Florida, EUA). The placebo mouthwash was formulated with the same components, except for CBD.

The tested CBD concentrations 0.01%, 0.05%, and 0.1% were selected based on the findings of Barak et al. (2022) [28], who reported a minimum inhibitory concentration (MIC) and minimum biofilm inhibitory concentration (MBIC) of 5 µg/mL against cariogenic species. These concentrations were chosen to encompass a range above this threshold and to allow the assessment of potential dose-dependent effects.

The pH of all formulations was maintained within the recommended range of 6–7. The positive control consisted of a commercially available 0.12% chlorhexidine mouthwash (Periogard, Colgate-Palmolive, São Bernardo do Campo, São Paulo, Brazil; pH = 4,74).

### Clinical phase and experimental groups

Each participant received one intraoral appliance per treatment condition, which were evaluated in a randomized order. For each experimental phase, participants wore the intraoral appliance for 7 days, followed by a 7-day washout period. After each washout interval, treatment allocation was determined by randomization performed by a collaborating researcher. The mouthwashes were dispensed in coded containers without product identification to ensure the double-blind design of the study. The experimental treatments consisted of five mouthwashes: a placebo (PL; negative control), a 0.12% chlorhexidine mouthwash (CHX; positive control), and three cannabidiol-based formulations containing 0.01% (CBD0.01%), 0.05% (CBD0.05%), and 0.1% (CBD0.1%) cannabidiol.

### In situ treatment protocol and cariogenic challenge

Three times daily, the intraoral devices were treated with the assigned mouthwash solution, simulating mouthwash use after the three main daily meals. This regimen was selected to reproduce a standardized daily exposure pattern consistent with regular adjunctive use of mouthwashes in oral hygiene routines. Although adherence to oral hygiene practices varies among individuals, and mouthwash is not necessarily used after every toothbrushing episode, some patients may use mouthrinses after meals or as an adjunctive measure when toothbrushing is not performed. Therefore, the three-times-daily protocol was adopted as a clinically plausible condition to evaluate the effects of repeated exposure to the experimental formulations under controlled in situ conditions. For this purpose, the appliances were removed from the oral cavity prior to meals. While outside the oral cavity, the appliances were stored in containers containing moistened gauze to prevent specimen dehydration.

The appliances were immersed in the assigned treatment solution for 30 s, ensuring complete immersion of the device. The bovine enamel specimens were not brushed and were exposed only to the immersion protocol. After treatment, the appliances were reinserted into the oral cavity. Participants were instructed to wait at least 15 min after meals before reinserting the appliance. The treatment protocol was carried out for 7 consecutive days.

In addition to the treatment protocol, a cariogenic challenge was induced on one side of each appliance. Every 2 h (eight times daily), participants removed the appliance and applied a 20% sucrose solution onto each enamel specimen located on one designated side of the device [23, 29]. After sucrose application, the appliances were kept outside the oral cavity for 5 min to allow sucrose diffusion into the biofilm before reinsertion. The 20% sucrose solution was prepared and replaced every 3 days. Sucrose application was performed at time points different from those of the treatment immersion protocol.

After 7 days of treatment, on the morning of the 8th day, participants remained in a fasting state and returned the used intraoral appliances. The biofilm was subsequently collected by the researchers. The polyethylene mesh covering the enamel specimens was carefully removed, and the biofilm was harvested using plastic curettes, taking care not to damage the enamel surfaces. Following biofilm collection, the enamel fragments were removed from the appliances and gently cleaned with gauze and deionized water. The specimens were then subjected to surface roughness, Knoop microhardness, and color analyses, as previously described.

After completion of these analyses, a new 7-day washout period was initiated, during which participants did not wear the intraoral appliance. At the end of the washout period, new enamel specimens were inserted into the appliances to begin the subsequent experimental phase. This cycle was repeated until all five in situ phases were completed.

### Surface roughness and microhardness changes

Ra and KHN were evaluated before and after the proposed treatments. The change in surface roughness (ΔRa) was calculated as the difference between final and baseline values. The percentage change in KHN was calculated relative to baseline values using the following formula:

$$\:\mathrm{K}\mathrm{H}\mathrm{N}=\left(\left(\mathrm{K}\mathrm{H}\mathrm{N}\mathrm{f}\:-\mathrm{K}\mathrm{H}\mathrm{N}\mathrm{i}\right)/\mathrm{K}\mathrm{H}\mathrm{N}\mathrm{i}\right)\times\:100$$

Where:

KHNi corresponds to baseline microhardness values, and.

KHNf corresponds to final microhardness values after treatment.

### Color change evaluation

L*, a*, and b* values were recorded for each specimen before and after treatment. Color differences were calculated using the CIEDE2000 formula (ΔE_00_):

$$\:\varDelta\:\mathrm{E}00=(\mathrm{L}/\mathrm{K}\mathrm{L}.\mathrm{S}\mathrm{L})+{(\mathrm{C}/\mathrm{K}\mathrm{C}.\mathrm{S}\mathrm{C})}^{2}\:+{(\mathrm{H}/\mathrm{K}\mathrm{H}.\mathrm{S}\mathrm{H})}^{2}+\mathrm{R}\mathrm{T}.(\mathrm{C}/\mathrm{K}\mathrm{C}.\mathrm{S}\mathrm{C})\times\:{(\mathrm{H}/\mathrm{K}\mathrm{H}.\mathrm{S}\mathrm{H})}^{0.5}$$

Where:

ΔL′, ΔC′, and ΔH′ represent differences in lightness, chroma, and hue between two measurements;

RT (rotation function) accounts for the interaction between chroma and hue differences in the blue region;

SL, SC, and SH are weighting functions for lightness, chroma, and hue;

KL, KC, and KH are parametric factors related to viewing conditions, which were set to 1 [30].

### Biofilm bacterial and yeast analysis

On the eighth day, after removal of the intraoral appliances, samples were placed in Stuart medium (MERCK, Darmstadt, Hesse, Germany) and transported under refrigeration (4 °C) to the laboratory on the same day for microbiological analysis. The assay was conducted according to Martins et al. (2011) [31], with modifications. Samples were transferred to Eppendorf-type tubes containing phosphate-buffered saline (PBS; pH 7.4). The tubes were subjected to ultrasonic bath treatment (SolidSteel, Piracicaba, SP, Brazil) for 30 min, followed by vortex agitation (Labnet, Edison, NJ, USA) for 1 min to promote homogeneous biofilm dispersion. Subsequently, 20 µL of the suspension were added to 180 µL of Brain Heart Infusion (BHI) broth (Kasvi, Pinhais, PR, Brazil) in 96-well microplates to perform serial dilutions ranging from 10^− 1^ to 10^− 7^. Aliquots of 50 µL from the original suspension (10^0^) and from each dilution 10^− 1^ to 10^− 7^ were plated onto Columbia agar plates (Difco, Sparks, MD, USA) supplemented with 5% defibrinated horse blood, divided into eight Sect [32]., and incubated at 37 °C for 24 h to promote the growth of fastidious and non-fastidious bacteria. The same procedure was performed using CHROMagar™ Candida (Difco) plates, incubated at 37 °C for 48 h for yeast growth and differentiation. After incubation, colony-forming units per milliliter (CFU/mL) were quantified. CFU/mL counts were subsequently log₁₀-transformed prior to statistical analysis to normalize data distribution.

### Enamel surface characterization by scanning electron microscopy

Enamel surface morphology was characterized using scanning electron microscopy (SEM; JSM-6610LV, JEOL, Tokyo, Japan). Specimens were initially dehydrated in a desiccator containing silica gel for 12 h at room temperature. Subsequently, the samples were mounted on aluminum stubs using double-sided conductive carbon tape (Electron Microscopy Sciences, Washington, PA, USA). All specimens were sputter-coated with a gold–palladium alloy (SCD 050 sputter coater, Bal-Tec, Balzers, Liechtenstein) to render the surface electrically conductive. The samples were examined under the scanning electron microscope at magnifications of 1000×, 2000×, and 5000×, or higher when necessary to visualize specific surface details. Imaging was performed at an accelerating voltage of 20 kV, with a working distance (WD) of 25 mm and a probe current (spot size) ranging from 25 pA to 100 pA.

### Statistical analysis

Data were initially tested for normality using the Shapiro–Wilk test and for homogeneity of variances using Levene’s test (α = 0.05). Ra, KHN, and ΔE_00_ data met the assumptions of normality and homoscedasticity. Because the study followed a crossover in situ design, in which all participants received all experimental treatments, the repeated-measures structure of the data was considered in the statistical model. Treatment and time were included as fixed factors, and participants were treated as the repeated-measures factor to account for within-subject correlation. The treatment × time interaction was tested for all response variables. When the interaction was not significant, the main effects of treatment and time were interpreted. When significant main effects were detected, pairwise comparisons were performed using Bonferroni’s post hoc test. The significance level was set at 5%.

Biofilm and yeast data did not meet the assumptions of normality and homoscedasticity and were analyzed using the non-parametric Kruskal–Wallis test, followed by Dunn’s post hoc test. These data were expressed as median (minimum–maximum), with a significance level set at *p* < 0.05. All statistical analyses were performed using GraphPad Prism software (GraphPad Software Inc., San Diego, CA, USA).

## Results

### Surface roughness

Surface roughness (ΔRa) results are presented in Table 1. No significant effect of sucrose exposure was observed on surface roughness changes (*p* > 0.05). Regarding treatment effects, a significant increase in roughness was observed only for the CBD 0.1% group compared with chlorhexidine (CHX) under sucrose conditions (*p* < 0.05). No statistically significant differences were found among the remaining groups (*p* > 0.05).

**Table 1:** Results surface roughness (ΔRa, µm) 2-way ANOVA (Bonferroni *p*<0.05)

	Placebo	CBD 0.01%	CBD 0.05%	CBD 0.1%	CHX
Sucrose-exposed	0.15 (0.12) abA	0.13 (0.10) abA	0.15 (0.06) abA	0.22 (0.22) aA	0.09 (0.05) bA
Non-Sucrose	0.15 (0.08) aA	0.16 (0.10) aA	0.13 (0.06) aA	0.13 (0.08) aA	0.12 (0.04) aA

Different letters, lowercase in the row and uppercase in the column, indicate statistically different results (*p*<0.05)

### Knoop microhardness

The relative microhardness (%) results are shown in Table 2. A significant effect of sucrose exposure was observed for the Placebo and CBD 0.01% groups, with specimens exposed to sucrose showing greater reduction in microhardness compared to baseline (*p* < 0.05). No statistically significant differences were found among treatments, regardless of sucrose exposure (*p* > 0.05).

**Table 2:** Results knoop microhardness(%ΔKHN) (%) – 2-way ANOVA (Bonferroni, *p* < 0.05)

	Placebo	CBD 0,01%	CBD 0,05%	CBD 0,1%	CHX
Sucrose-exposed	-46.04 (9.82) aA	-36.6 (10.40) aA	-37.96 (10.83) aA	-36.85 (8.97) aA	-42.38 (11.00) aA
Non- Sucrose	-28.24 (20.55) aB	-21.00 (5.76) aB	-27.43 (11.78) aA	-33.25 (13.4) aA	-33.205 (13.00) aA

Different letters, lowercase in the row and uppercase in the column, indicate statistically different results (*p*<0.05).

### Color change (ΔE_00_)

**Fig. 2:**
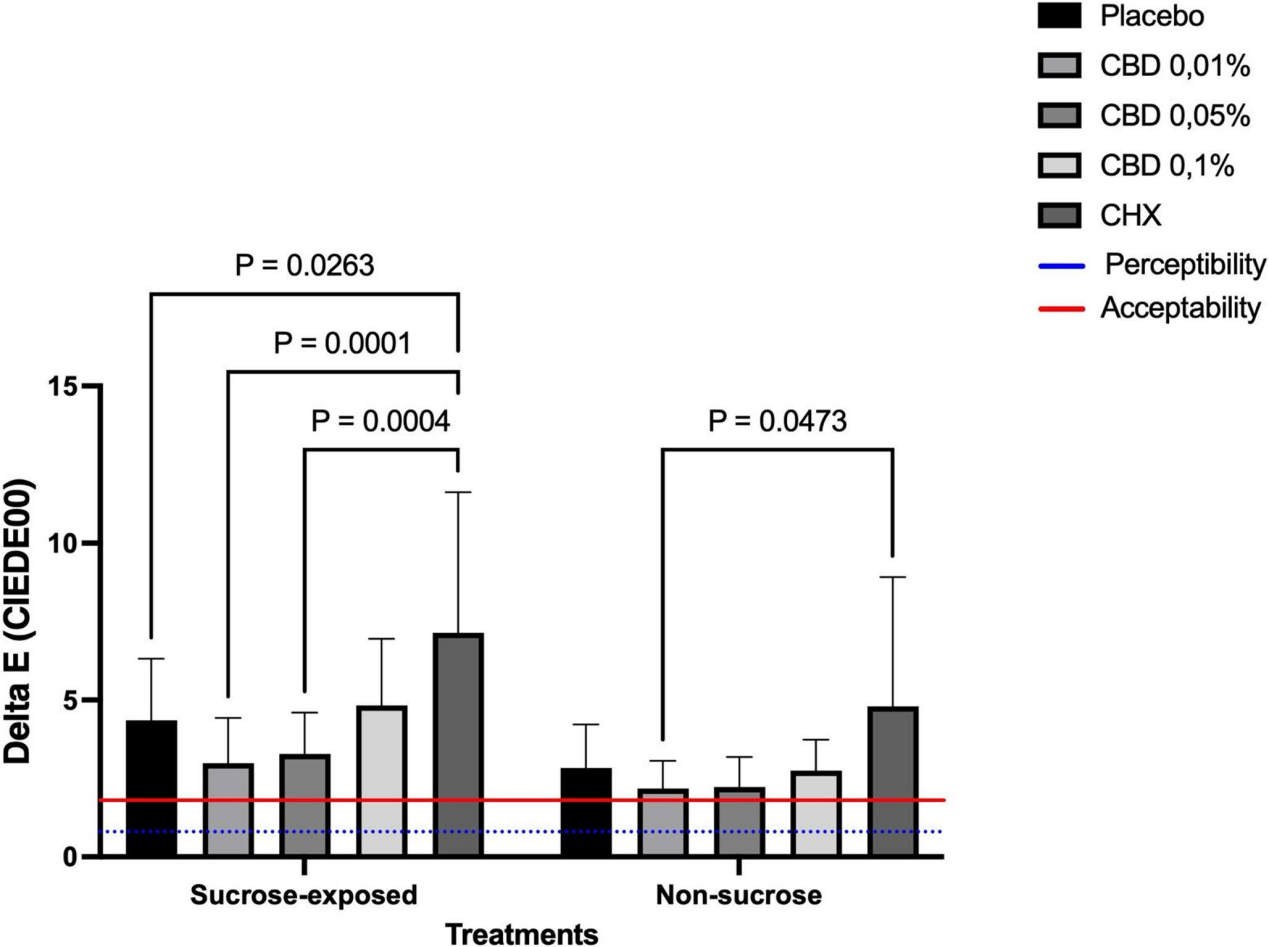
Color change (ΔE_00_) of enamel specimens after treatment with different mouthwash under sucrose-exposed and non-sucrose conditions. The groups evaluated were Placebo, CBD 0.01%, CBD 0.05%, CBD 0.1%, and CHX. Horizontal lines indicate perceptibility (0.8) and acceptability (1.8) thresholds. The figure was created using GraphPad Prism (GraphPad Software Inc., San Diego, CA, USA)

Color change results are presented in Fig. 2. Two-way ANOVA revealed no significant interaction between treatment and sucrose exposure (*p* > 0.05), and sucrose did not significantly influence ΔE_00_ values. However, treatment had a significant effect on color change. Under sucrose conditions, CHX showed the highest ΔE_00_ values, significantly different from the other groups, except for CBD 0.1% (*p* < 0.05). In the absence of sucrose, CHX again showed the highest color change, significantly different from CBD 0.01%, which presented the lowest ΔE_00_ values (*p* < 0.05). No significant differences were observed among the remaining groups (*p* > 0.05).

All ΔE_00_ values exceeded the perceptibility (0.8) and acceptability (1.8) thresholds proposed by Paravina et al. (2015) [33], as illustrated in Fig. 2. Overall, higher ΔE_00_ values were observed under sucrose conditions, particularly for CHX, followed by CBD 0.1%. In the absence of sucrose, color changes were reduced but remained above the acceptability threshold.

### Biofilm analysis

Biofilm formation results (log CFU/mL) are presented in Table 3. Sucrose exposure did not significantly influence biofilm formation (*p* > 0.05). Under sucrose conditions, the CBD 0.01% group showed significantly higher biofilm formation compared to CHX (*p* < 0.05), with no differences compared to the other groups. CBD 0.05% and CBD 0.1% did not differ significantly from CHX (*p* > 0.05). In the absence of sucrose, CHX showed significantly lower biofilm levels compared to CBD 0.01% and CBD 0.1% (*p* < 0.05). No significant differences were observed among the remaining groups (*p* > 0.05).

**Table 3:** Biofilm (log₁₀ CFU/mL) –Kruskal-Wallis (Dunn, *p* < 0.05) –median (minimum–maximum values)

	Placebo	CBD 0.01%	CBD 0.05%	CBD 0.1%	CHX
Sucrose-exposed	6.8 (4.4 – 8.3) abA	7.1 (4.3 -8.8) bA	6.7 (3.8 – 9.9) abA	6.8 (3.0 – 8.2) abA	6.0 (4.4 – 8.9) aA
Non-Sucrose	6.1 (3.8 – 8.1) abA	6.5 (4.6 – 8.0) aA	6.2 (4.4 – 9.0) abA	6.8 (5.0 – 9.2) aA	5.4 (3.5 – 7.7) bA

Different letters, lowercase in the row and uppercase in the column, indicate statistically different results (*p*<0.05).

### Yeast analysis

Yeast counts (log CFU/mL) are presented in Table 4. Sucrose exposure did not significantly influence yeast growth (*p* > 0.05). CHX showed the lowest yeast counts regardless of sucrose presence, being significantly different from CBD 0.01% and CBD 0.1% (*p* < 0.05). The Placebo and CBD 0.05% groups showed intermediate values and did not differ significantly from each other or from CHX (*p* > 0.05).

**Table 4:** Yeast (log₁₀ CFU/mL) –Kruskal-Wallis (Dunn, *p* < 0.05) –median (minimum–maximum)

	Placebo	CBD 0.01%	CBD 0.05%	CBD 0.1%	CHX
Sucrose-exposed	1.1 (0 – 3.6) abA	1.8 (0.0 – 3.9) aA	1.5 (0.0 – 2.4) abA	1.8 (0.0 – 4.3) aA	0.3 (0.0 – 1.2) bA
Non-Sucrose	1.0 (0.0 – 3.1) abA	1.6 (0.0 – 3.8) bA	0.5 (0.0 – 1.9) abA	1.3 (0.0 – 3.6) bA	0.0 (0.0 – 1.0) aA

Different letters, lowercase in the row and uppercase in the column, indicate statistically different results (*p*<0.05).

### Scanning Electron Microscopy (SEM)

**Fig. 3:**
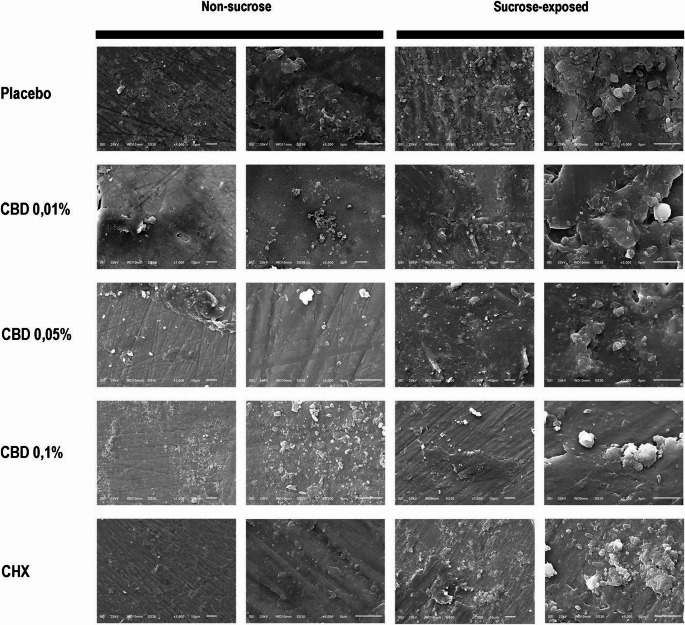
Scanning electron microscopy (SEM) images of enamel surfaces after treatment with different mouthwash. Representative micrographs were obtained at magnifications of 1000× and 5000× for the following groups: Placebo, CBD 0.01%, CBD 0.05%, CBD 0.1%, and CHX 0.12%, under conditions with and without sucrose exposure

SEM images (Fig. 3) revealed greater biofilm accumulation on specimens sucrose exposed, regardless of treatment, compared to that non-sucrose. Specimens treated with CHX exhibited less dense and less organized biofilm in the absence of sucrose, with visible polishing marks, similar to the CBD 0.05% group. CBD 0.01% and CBD 0.1% showed slightly higher biofilm accumulation compared to CHX. In contrast, the Placebo group exhibited a denser biofilm with greater matrix accumulation. Under sucrose conditions, only the CBD 0.1% group showed visible polishing marks, indicating reduced biofilm accumulation. All other groups, including CHX, presented biofilm coverage, although CHX showed lower accumulation compared to CBD 0.01% and Placebo.

## Discussion

The present in situ study evaluated the antibiofilm potential of cannabidiol-based mouthwash at different concentrations, as well as their effects on the physical and mechanical properties of dental enamel. This approach is particularly relevant, as in situ models allow the evaluation of biofilm dynamics and substrate interactions under clinically relevant conditions, including the influence of saliva and dietary factors [21]. The study was conducted based on the hypothesis that experimental mouthwashes containing cannabidiol, regardless of the concentration used, would not promote significant changes in the color, surface roughness, or microhardness of dental enamel compared to chlorhexidine, nor would they exhibit superior antimicrobial activity compared to the positive control.

Regarding surface roughness, the first hypothesis was rejected, as CBD 0.1% promoted greater changes compared to CHX under sucrose exposure. However, the exposure to sucrose did not significantly affect enamel surface roughness, since no statistically significant differences were observed between specimens with and without cariogenic challenge. Additionally, none of the experimental mouthwash or CHX differed from the Placebo. These findings are consistent with previous in vitro studies showing that mouthwashes, including chlorhexidine, do not significantly alter enamel surface roughness [34], and with in situ evidence that sucrose exposure for 7 days can promote measurable enamel changes [23], an effect not observed in most groups in the present study. Since both chlorhexidine and cannabidiol primarily act on the biofilm rather than directly interacting with the mineral structure of enamel [28, 35–37], these agents did not produce measurable changes in surface topography, supporting their safety regarding enamel integrity.

Microhardness analysis demonstrated that sucrose exposure significantly reduced enamel hardness in the Placebo and CBD 0.01% groups, reinforcing the well-established role of fermentable carbohydrates in driving demineralization processes [5]. In contrast, CBD 0.05% and CBD 0.1% performed similarly to chlorhexidine in maintaining enamel microhardness, suggesting a protective potential at higher concentrations.

Regarding color change (ΔE_00_), the highest values were observed for CHX, which differed significantly from the Placebo and the experimental mouthwash at concentrations of 0.01% and 0.05% under cariogenic challenge, and from CBD 0.01% in the absence of sucrose. These findings are consistent with the literature, which reports adverse effects associated with the prolonged use of CHX, including taste alteration, dental discoloration, and staining of teeth and tongue [9, 12–14]. Sucrose exposure did not significantly influence color change. However, for the experimental mouthwash, ΔE_00_ values were closer to the acceptability threshold (ΔE_00_ = 1.8; Paravina et al., 2015) [32] in the absence of sucrose compared to specimens subjected to cariogenic challenge.

Another relevant aspect of the present study was the evaluation of biofilm formation and the antibiofilm activity of the tested mouthwashes. Sucrose exposure did not significantly influence biofilm accumulation, which was unexpected given its well-established role as a primary substrate for biofilm formation due to its unique glycosidic structure and high energy content [5, 6, 38]. This may be explained by the frequency-dependent nature of sucrose-induced cariogenicity, whereby a saturation effect has been reported at high exposure frequencies [6, 39], as well as by the existence of a concentration threshold (~ 5% sucrose) above which no additional cariogenic effect is observed, and by the buffering capacity of saliva, which may have attenuated biofilm acidification and accumulation [40, 41].

Despite the lack of a sucrose effect, differences among treatments were observed. Under sucrose conditions, CHX showed lower biofilm formation compared to CBD 0.01%, confirming its well-established antimicrobial efficacy [34, 37]. In contrast, CBD at concentrations of 0.05% and 0.1% did not differ significantly from CHX, suggesting that higher cannabidiol concentrations may be sufficient to modulate biofilm formation.

Interestingly, the Placebo group showed a similar pattern to CHX and the higher CBD concentrations. This finding may be attributed to the presence of sodium benzoate in its formulation, a preservative with known antimicrobial activity capable of interfering with bacterial adhesion and biofilm formation [42, 43]. Thus, the placebo may not have behaved as a true negative control, acting instead as a modulator of biofilm formation.

SEM findings supported these results, showing reduced biofilm density and more exposed enamel surfaces in the CHX and CBD 0.05% groups, whereas denser and more organized biofilms were observed in the Placebo and CBD 0.01% groups. These observations are consistent with previous studies indicating that CHX reduces bacterial viability and extracellular polysaccharide synthesis, resulting in a less structured biofilm without complete matrix removal [44, 45].

Although cannabidiol has demonstrated antimicrobial and antibiofilm activity against oral pathogens in previous studies [28, 46, 47], its effects appear to be concentration-dependent. In the present study, CBD 0.01% showed limited efficacy, whereas higher concentrations exhibited a biofilm-modulating effect that performed similarly to CHX. This is consistent with its proposed mechanism of action, which involves disruption of bacterial membranes, increased permeability, inhibition of extracellular polysaccharide synthesis, and reduced bacterial adhesion [28, 36].

Additionally, cannabidiol appears to act through a more gradual and multifactorial mechanism compared to CHX. While CHX promotes rapid bacterial disruption and exhibits high substantivity, cannabidiol may exert progressive effects on biofilm structure and organization. Although limited data are available regarding its substantivity, previous findings suggest that its antimicrobial activity may persist due to interactions with the acquired pellicle and oral tissues [48].

Finally, it should be noted that biofilm evaluation in this study was based on quantitative analysis only. This methodological approach may have limited the detection of important qualitative changes, such as reduced cohesion, structural disorganization, or alterations in biofilm architecture, which have been reported in the literature for cannabidiol [28].

Regarding yeast quantification, the Placebo group did not differ significantly from CHX or cannabidiol-treated groups (0.01%, 0.05%, and 0.1%). This finding may be explained by the presence of sodium benzoate in the placebo formulation, which has well-documented antifungal activity [42, 49]. This compound can inhibit yeast growth by disrupting cellular metabolism and promoting intracellular acidification, potentially preventing the placebo from acting as a fully inert control.

As expected, CHX showed low yeast counts due to its well-established antifungal activity, which involves rapid disruption of the cytoplasmic membrane, leakage of intracellular components, and high substantivity on dental surfaces [50, 51]. In contrast, CBD 0.01% presented significantly higher yeast counts, likely due to its low concentration, which may be insufficient to induce membrane disruption or oxidative stress in fungal cells.

Previous studies have demonstrated that cannabidiol exhibits antifungal and antibiofilm activity against *Candida albicans*, including inhibition of biofilm formation and disruption of established biofilms [52, 53]. These effects are associated with interference in membrane integrity, reduction of extracellular polysaccharide production, induction of oxidative stress, and modulation of virulence-related pathways.

In the present study, CBD at concentrations of 0.05% and 0.1% did not differ from CHX, suggesting that higher concentrations may achieve similar antifungal effects. This is consistent with evidence showing that cannabidiol can reduce extracellular polysaccharide production, alter biofilm architecture, inhibit yeast-to-hypha transition, increase oxidative stress, and promote fungal cell death in a concentration-dependent manner [54].

Thus, beyond reducing yeast counts, cannabidiol may act as a modulator of fungal biofilm organization and maturation, contributing to matrix disruption and increasing susceptibility to antimicrobial agents and mechanical removal. This multifactorial mechanism, involving membrane permeability, metabolic disruption, and biofilm interference, may also reduce the likelihood of resistance development compared to conventional agents such as chlorhexidine, supporting its potential as an alternative for the control of oral fungal biofilms.

Despite the methodological rigor inherent to an in situ study design, one limitation should be acknowledged. The placebo mouthwash contained sodium benzoate, an antimicrobial preservative included to ensure the microbiological stability and shelf life of the formulation, which is a standard practice in the compounding of aqueous mouthwash preparations [55]. Importantly, sodium benzoate is also a recognized excipient in 0.12% chlorhexidine mouthwash formulations, which served as the positive control in the present study [55, 56]. Therefore, any antimicrobial effect potentially attributable to sodium benzoate would have been present in both the placebo and positive control groups. As such, this compound did not confer an exclusive advantage to the placebo group, nor did it compromise the validity of the between-group comparisons.

Another limitation of the present study is that an a priori power analysis was not performed. The sample size was defined based on previous in situ studies with similar crossover designs and comparable outcome variables. Although this approach is commonly adopted in exploratory in situ studies, future investigations should include an a priori sample size calculation based on the primary outcome to strengthen the statistical planning.

## Conclusion

Based on the findings of this study and considering its limitations, it can be concluded that cannabidiol-based mouthwashes were able to modulate dental biofilm formation in a concentration-dependent manner, with higher concentrations (0.05% and 0.1%) demonstrating performance similar to chlorhexidine in reducing biofilm accumulation, influencing its structural organization, and maintaining relative microhardness. This effect did not result in alterations to enamel surface topography. However, cannabidiol-based mouthwashes maintained color closer to acceptability thresholds.

## Data Availability

The data that support the findings of this study are available from the corresponding author upon reasonable request.
